# Hospital-acquired colonization and infections in a Vietnamese intensive care unit

**DOI:** 10.1371/journal.pone.0203600

**Published:** 2018-09-07

**Authors:** Duong Bich Thuy, James Campbell, Le Thanh Hoang Nhat, Nguyen Van Minh Hoang, Nguyen Van Hao, Stephen Baker, Ronald B. Geskus, Guy E. Thwaites, Nguyen Van Vinh Chau, C. Louise Thwaites

**Affiliations:** 1 Hospital for Tropical Diseases, Wellcome Trust Asia Programme, Oxford University Clinical Research Unit (OUCRU), Ho Chi Minh City, Vietnam; 2 Adult Intensive Care Unit, Hospital for Tropical Diseases, Ho Chi Minh City, Vietnam; 3 Centre for Tropical Medicine and Global Health, Nuffield Department of Clinical Medicine, Oxford University, Oxford, United Kingdom; 4 Department of Infectious Diseases, University of Medicine and Pharmacy, Ho Chi Minh City, Vietnam; 5 Department of Medicine, Cambridge University, Cambridge, United Kingdom; 6 Board of Directors, Hospital for Tropical Diseases, Ho Chi Minh City, Vietnam; Medical University of Gdansk, POLAND

## Abstract

Data concerning intensive care unit (ICU)-acquired bacterial colonization and infections are scarce from low and middle-income countries (LMICs). ICU patients in these settings are at high risk of becoming colonized and infected with antimicrobial-resistant organisms (AROs). We conducted a prospective observational study at the Ho Chi Minh City Hospital for Tropical Diseases, Vietnam from November 2014 to January 2016 to assess the ICU-acquired colonization and infections, focusing on the five major pathogens in our setting: *Staphylococcus aureus* (*S*. *aureus*), *Escherichia coli* (*E*. *coli*), *Klebsiella* spp., *Pseudomonas* spp. and *Acinetobacter* spp., among adult patients with more than 48 hours of ICU stay. We found that 61.3% (223/364) of ICU patients became colonized with AROs: 44.2% (161/364) with rectal ESBL-producing *E*. *coli* and *Klebsiella* spp.; 30.8% (40/130) with endotracheal carbapenemase-producing *Acinetobacter* spp.; and 14.3% (52/364) with nasal methicillin-resistant *S*. *aureus*. The incidence rate of ICU patients becoming colonized with AROs was 9.8 (223/2,276) per 100 patient days. Significant risk factor for AROs colonization was the Charlson Comorbidity Index score. The proportion of ICU patients with HAIs was 23.4% (85/364), and the incidence rate of ICU patients contracting HAIs was 2.3 (85/3,701) per 100 patient days. The vascular catheterization (central venous, arterial and hemofiltration catheter) was significantly associated with hospital-acquired bloodstream infection. Of the 77 patients who developed ICU-acquired infections with one of the five specified bacteria, 44 (57.1%) had prior colonization with the same organism. Vietnamese ICU patients have a high colonization rate with AROs and a high risk of subsequent infections. Future research should focus on monitoring colonization and the development of preventive measures that may halt spread of AROs in ICU settings.

## Introduction

Hospital-acquired infections (HAIs) are a major global health problem, with World Health Organization (WHO) estimating that millions of patients are affected each year [1]. Critically ill patients are particularly vulnerable and infection rates are approximately double those of other patients [2]. Although data from resource-limited settings are sparse, infection rates are likely higher than in high-income settings [3–5].

Prior bacterial colonization may increase the risk of subsequent HAIs in ICU. Many studies have investigated the relationship between colonization on admission to ICU and subsequent infection. Admission colonization with *S*. *aureus* has been shown to be a significant risk factor for ICU-acquired *S*. *aureus* infections [6,7]. Similarly, rectal carriage of multidrug-resistant *Enterobacteriaceae* on ICU admission has been identified as a key factor for subsequent bloodstream, lung, urinary tract, and central venous catheter infection [8,9]. Whilst the majority of these studies only assessed colonization and infection with the same bacterial species according to their antimicrobial susceptibility profile, a study conducted in a Spanish ICU found that among 77 patients who developed *Pseudomonas aeruginosa* (*P*. *aeruginosa*) pneumonia, 69 (89.6%) had prior *P*. *aeruginosa* rectal colonization, and 60 (87.0%) of these paired rectal and clinical isolates exhibited genotyping concordance [10]. In addition to colonization on admission, ICU patients acquired bacterial colonization which also may lead to subsequent HAIs. A study of 189 consecutive patients in another Spanish ICU found that 20 (10.6%) patients were colonized with multidrug-resistant *Acinetobacter baumannii* (*A*. *baumannii*) upon ICU admission, and 57 (30.2%) additional patients acquired colonization, mostly during the first week of ICU admission. Rectal colonization was associated with increased HAIs with multidrug-resistant *A*. *baumannii* [11].

Understanding colonization and its relationship to HAIs is central for providing the necessary data to ensure safe and high-quality healthcare. In LMICs, such as Vietnam, there is a large amount of antimicrobial use in the community [12,13]. In our previous publication, we found that up to 63.1% (529/838) patients colonized with AROs on ICU admission [14]. Due to a paucity of antimicrobial stewardship programs, the situation of colonization, infection and antimicrobial resistance may be different and more serious in our country. To conserve scarce resources and tailor infection control programs appropriately, this relationship needs to be better understood. We performed a year-long prospective observational study examining acquired colonization and HAIs in a medical ICU at the Ho Chi Minh City Hospital for Tropical Diseases, Vietnam, focusing on the five major pathogens in our setting: *S*. *aureus*, *E*. *coli*, *Klebsiella* spp. (including *K*. *pneumoniae)*, *Pseudomonas* spp. (including *P*. *aeruginosa)*, and *Acinetobacter* spp. (including *A*. *baumannii)*.

## Materials and methods

### Setting

We conducted a prospective observational study in Adult ICU of the Ho Chi Minh City Hospital for Tropical Diseases, a tertiary referral hospital for infectious diseases serving Southern Vietnam. Adult ICU is a 20-bed medical ward, but usually admits additional patients. There are approximately 24–28 patients per day and 1,000–1,200 admissions per year. The nurse-to-patient ratio is about 1:3, and varying numbers of nursing students may also be present and take part in clinical activities. The ICU is divided into 4 small blocks (about 4–8 patients per block). The standard infection control measures are in place, including personal protective equipment for routine patient care (head coverings, mask, and gloves); daily patient bathing with 2% chlorhexidine gluconate; healthcare worker education and adherence monitoring with a focus on hand hygiene. The mean rate of hand hygiene compliance in Adult ICU is about 70–80%. Anios Special DJP SF (Laboratoires Anios ^TM^, France) is used for disinfection of ICU surfaces by air, and Surfa’Safe (Laboratoires Anios ^TM^, France), detergent disinfectant foam for non-invasive medical device surfaces after patient discharge. The surveillance of HAIs has been a mandatory action in our ICU since 2015, with mean prevalence of 5–10%. The most frequent types of HAIs were pneumonia, urinary tract infection (UTI) and bloodstream infection (BSI). The surgical site infections are extremely rare in Adult ICU because Adult ICU is dedicated to management of critically-ill patients with severe infectious diseases like tetanus, Dengue infection, influenza, sepsis or septic shock, and end-stage cirrhosis. Moreover, active screening for patients with AROs is not available in our unit. Therefore, contact isolation or geographic separation of colonized or infected patients with AROs is rarely applied.

The study was reviewed and approved by the Ethics Committee of the Ho Chi Minh City Hospital for Tropical Diseases, Vietnam and the Oxford Tropical Research Ethics Committee (OxTREC), United Kingdom.

### Study population and procedure

All patients aged ≥15 years admitted to ICU with an expected length of stay of >48 hours between 10th November 2014 and 14th January 2016 were eligible for entry to the study. Written informed consent was obtained from all the participants or their representatives. All patients admitted to ICU within 90 days from an earlier discharge were excluded. Nasal swab, rectal swab, and/or endotracheal aspirate (in case of intubation or tracheostomy) were taken for detecting acquired colonization with *S*. *aureus*, *E*. *coli*, *Klebsiella* spp., *Acinetobacter* spp. and *Pseudomonas* spp. and subsequent HAIs. Swabs were taken within 48 hours of ICU admission and repeated twice a week (on Monday and Thursday) until patients were discharged from ICU.

### Microbiological methods

Nasal and endotracheal specimens were cultured on Blood agar (bioMérieux) and MacConkey (bioMérieux) to specifically isolate *S*. *aureus*, *E*. *coli*, *Klebsiella* spp., *Pseudomonas* spp. and *Acinetobacter* spp.. Staphylococcal colonies were checked for catalase and coagulase, plus checked for methicillin resistance using cefoxitin disc diffusion, and re-checked on the matrix assisted laser desorption/ionization time-of-flight mass spectrometry (MALDITOF, Bruker) [14].

Xylose Lysine Deoxycholate agar (bioMérieux) and MacConkey agar (bioMérieux) were used to culture rectal swabs for Gram negative bacteria, and identification of *E*. *coli*, *Klebsiella* spp., *Pseudomonas* spp. and *Acinetobacter* spp. was confirmed by MALDITOF. Antimicrobial susceptibility testing was conducted by the Kirby/Bauer disc diffusion method and interpreted using the Clinical and Laboratory Standards Institute guidelines 2015 [15]. Appropriate organisms were screened using CHROMagar (CHROMagar, Paris, France) for ESBL production. The double disc diffusion method was then used to detect ESBL activity using both cefotaxime and ceftazidime, alone and in combination with clavulanate. ESBL activity is considered if there is a ≥ 5mm increase in a zone diameter for either antimicrobial agent tested in combination with clavulanate compared to the zone diameter of the agent when tested alone. CHROMagar C3G (CHROMagar, Paris, France) was used as a screening agar for AmpC production. Then suitable colonies had an AmpC induction test to detect induced AmpC lactamase activity. A ceftazidime disc was placed near cefoxitin/imipenem. A flattening zone of 3^rd^ cephalosporin toward the inducer (cefoxitin/imipenem) indicates the inducible AmpC lactamase. Moreover, ffollowing detection of reduced susceptibility to meropenem in routine susceptibility tests, a modified carbapenem inactivation method (mCIM) was performed to identify the production of carbapenemase. If the test isolate produces a carbapenemase, the meropenem in the disk will be hydrolyzed and there will be no inhibition or limited growth inhibition of the meropenem-susceptible E. coli ATCC® 25922 (the indicator organism).

Other clinical specimens (blood, urine and sputum) were cultured according to the Standard Operating Procedure of the Microbiology Department of the Ho Chi Minh City Hospital for Tropical Diseases.

### Definitions

For the purposes of this investigation, acquired colonization was defined by a positive surveillance culture with *S*. *aureus*, *E*. *coli*, *Klebsiella* spp., *Pseudomonas* spp. and/or *Acinetobacter* spp. preceded by a negative culture on admission or a positive surveillance culture with differently specified bacteria or with the same organisms but different resistance pattern compared to admission culture. Antimicrobial-resistant organisms (AROs) colonization was defined as a positive culture from nasal, rectal and/or endotracheal sample of methicillin-resistant *S*. *aureus*, 3^rd^-generation cephalosporin-resistant *E*. *coli*, 3^rd^-generation cephalosporin-resistant *Klebsiella* spp., ceftazidime-resistant *Pseudomonas* spp., carbapenem-resistant *E*. *coli*, carbapenem-resistant *Klebsiella* spp., carbapenem-resistant *Pseudomonas* spp. and/or carbapenem-resistant *Acinetobacter* spp.. AROs acquisition was defined by a positive surveillance culture with any of AROs preceded by negative culture(s) or a positive surveillance culture with differently specified AROs compared to previous cultures. An episode of HAIs was defined as a new infection, including pneumonia, bloodstream infection and urinary tract infection, that occurred 48 hours after ICU admission. Pneumonia, bloodstream infection (BSI) and urinary tract infection (UTI) were defined according to the criteria established by the Centers for Disease Control and Prevention, Atlanta, United States, 2014 [16].

### Statistical analysis

#### Analysis of risk factors for acquired colonization

Risk factors for specifically acquired nasal/rectal/endotracheal colonization with AROs during ICU stay were evaluated using Cox proportional cause-specific hazards regression, with discharge and death as competing events. The following possible risk factors on ICU admission were investigated: tetanus disease, Charlson Comorbidity Index score, colonization status (by anatomical sites), receipt of antimicrobial treatment, and intensive care procedures including nasogastric tube and respiratory support consisting of intubation, tracheostomy and mechanical ventilation. Respiratory support was tested for nasal and endotracheal (not for rectal) colonization with AROs. Tetanus disease was included in the model because the pathogenesis of tetanus disease is related to the activity of a neuro-toxin released from *Clostridium tetani* [17], which is different from other illnesses, of which the pathology is related to the host response to an infectious or non-infectious agent leading to systemic inflammatory, organ dysfunction and organ failure [18,19]. Moreover, due to a high proportion of tetanus patients (>56%, 204/364) in this study, tetanus disease was likely to act as a confounding variable for the other risk factors. The other variables were selected based on literature review [20–28]. The continuous variable Charlson Comorbidity Index score was included as linear term. All other factors were binary variables. It was assumed that patients remained acquired colonization with AROs from detection of AROs acquisition until ICU discharge.

#### Analysis of risk factors for HAIs development during ICU stay

Cox proportional hazards regression was also used to determine the risk factors for specific types of HAIs: pneumonia, BSI and UTI, and only the first episode of HAIs caused by any of the specified bacteria was included for analysis. However, multivariate Cox regression model was not performed for BSI due to a very low number of events. The following risk factors were considered: admission for tetanus disease, Charlson Comorbidity Index score, prior colonization status (including initial colonization on ICU admission and acquired colonization during ICU stay per anatomical sites), and intensive care procedures (respiratory support consisting of intubation, tracheostomy and mechanical ventilation for pneumonia; urinary catheter for UTI; and vascular catheters including central venous, arterial and hemofiltration catheter for BSI). In this model, prior nasal/rectal/endotracheal colonization is considered as a time-dependent risk factor in subsequent HAIs. Time zero was the date of ICU admission, and the date of acquired colonization was assumed to be at the midway point between the latest negative culture and the first positive surveillance culture. It was assumed that patients remained colonized from detection of colonization on ICU admission or acquisition during ICU stay until ICU discharge.

Statistical analyses used the R 3.4.0 software (R foundation, Vienna, Austria), especially the R functions *coxph* from the *survival* package. P values < 0.05 (two-sided) were considered statistically significant.

## Results

### General patient characteristics

**Fig 1:**
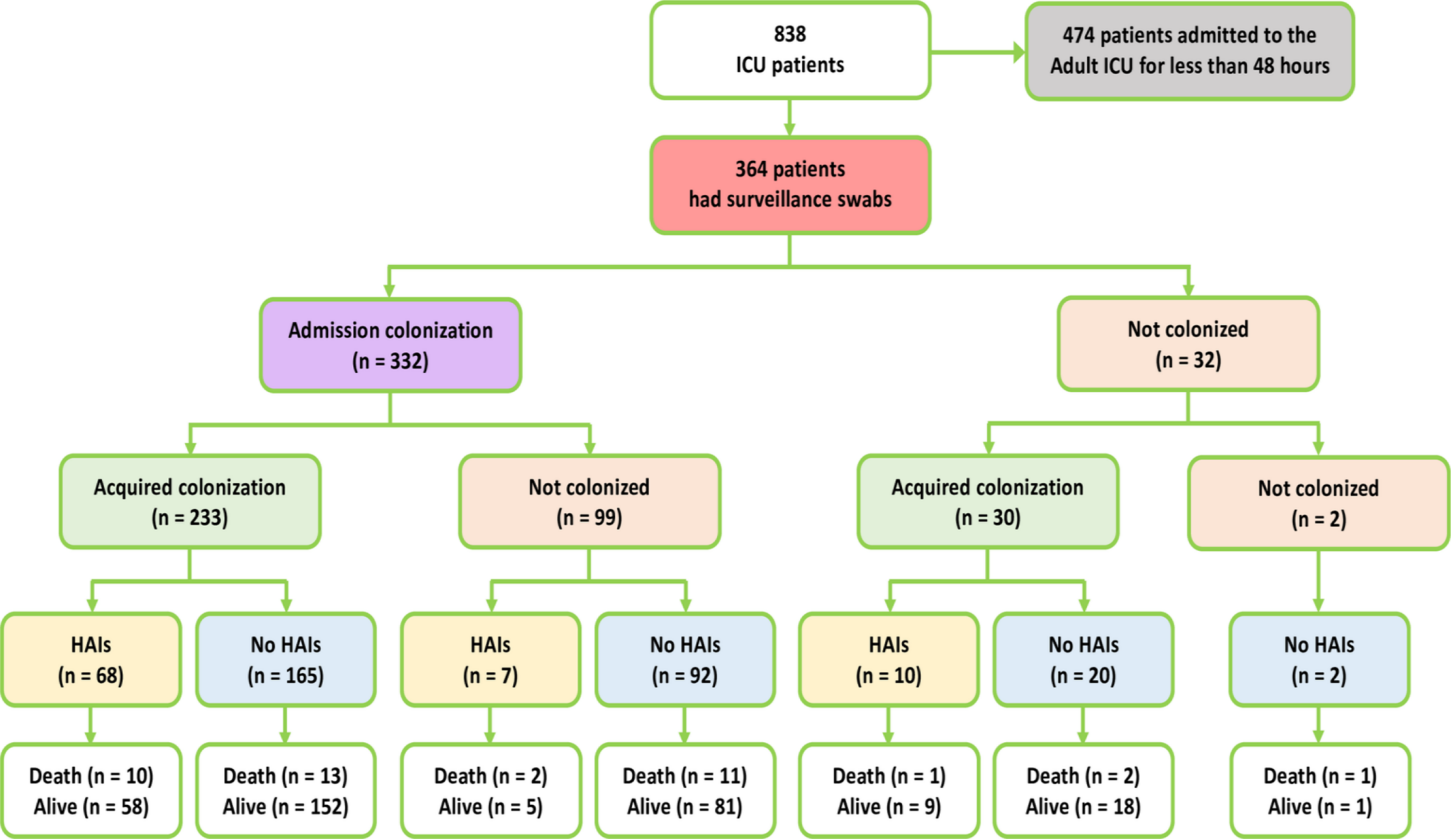
Inclusion of patients, taking surveillance swabs, and outcomes.

838 adults were enrolled in the study, of whom 364 were admitted for >48 hours and were screened for acquired colonization and HAIs (Fig 1). Of note is the large proportion of patients in this study with tetanus (56.0%, 204/364). The patient characteristics are described in Table 1.

**Table 1:** General patient characteristics during ICU stay.

Age (yr)–median (IQR)	46 (33–60)
**< 60 –**n (%)	265 (72.8)
**≥ 60 –**n (%)	99 (27.2)
**Sex**–n (%)	
**Male**	242 (66.5)
**Female**	122 (33.5)
**Charlson Comorbidity Index score–**median (IQR)	0 (0–1)
**No comorbidity (0)–**n (%)	247 (67.8)
**Mild (1–2)–**n (%)	73 (20.1)
**Moderate (3–4)–**n (%)	24 (6.6)
**Severe (≥ 5)–**n (%)	20 (5.5)
**APACHE II score–**median (IQR)	8 (3–14)
**Mild (< 5)–**n (%)	118 (32.4)
**Moderate (5–12)–**n (%)	134 (36.8)
**Severe (> 12)–**n (%)	112 (30.8)
**Admitting diagnosis–**n (%)	
**Tetanus**	204 (56.0)
**Sepsis and septic shock**	75 (20.6)
**Local infections** *	45 (12.4)
**Dengue infection**	17 (4.7)
**Internal medicine diseases** ^#^	23 (6.3)
**Death–**n (%)	**40 (11.0)**
**ICU stay (days)–**median (IQR)	**10 (5–18)**
**Hospital stay (days)–**median (IQR)	**22 (13–32)**

* Local infections included pneumonia (25 cases), cellulitis (4 cases), UTI (15 cases), and spontaneous bacterial peritonitis (1 cases)

# Internal medicine diseases included kidney failure (6 cases), myocarditis (3 cases), myocardial infarction (6 cases), atrial fibrillation (3 cases), malignant hypertension (2 cases), diabetic ketoacidosis (2 cases) and epilepsy (2 cases).

### Acquired colonization status

In this study, a total of 3,162 surveillance swabs were taken: 1,276 nasal swabs, 1,276 rectal swabs, and 610 endotracheal samples from 130 patients with endotracheal tubes in place. A total of 3,836 bacterial isolates were cultured, of which 378 (9.9%) were *S*. *aureus*, 1,550 (40.4%) were *E*. *coli*, 895 (23.3%) were *Klebsiella* spp., 555 (14.5%) were *Acinetobacter* spp., and 458 (11.9%) were *Pseudomonas* spp.. Their antimicrobial resistance characteristics are summarized in Table 2.

**Table 2:** Antimicrobial resistance of colonized bacteria (in percentage).

Antimicrobials n (%)	*S*.*aureus* (n = 378)	*E*.*coli* (n = 1,550)	*Klebsiella* spp. (n = 895)	*Acinetobacter* spp. (n = 555)	*Pseudomonas* spp. (n = 458)
Amoxcillin-clavulanic acid		924 (59.6)	260 (29.1)		
Ceftazidime		881 (56.8)	256 (28.6)	539 (97.1)	101 (22.1)
Ceftriaxone		881 (56.8)	256 (28.6)	539 (97.1)	
Cefepime		811 (52.3)	213 (23.8)	539 (97.1)	87 (19.0)
Ticarcillin-clavulanate		984 (63.5)	277 (30.9)	537 (96.8)	330 (72.1)
Piperacillin-tazobactam		815 (52.6)	219 (24.5)	537 (96.8)	81 (17.7)
Ofloxacin		767 (49.5)	131 (14.6)		
Ciprofloxacin	227 (60.1)	780 (50.3)	183 (20.4)		
Levofloxacin	221 (58.5)			352 (63.4)	56 (12.2)
Sulfamethoxazole-trimethoprim	19 (5.0)	1,102 (71.1)	335 (37.4)	302 (54.4)	438 (95.6)
Amikacin		25 (1.6)	27 (3.0)	215 (38.7)	23 (5.0)
Gentamycin					23 (5.0)
Ertapenem		42 (2.7)	32 (3.6)		
Imipenem		33 (2.1)	32 (3.6)	371 (66.8)	202 (44.1)
Meropenem		26 (1.7)	32 (3.6)	371 (66.8)	183 (40.0)
Colistin		27 (1.7)	44 (4.9)	6 (1.1)	0
Penicillin	368 (97.4)				
Oxacillin	275 (72.8)				
Vancomycin	0				
Erythromycin	265 (70.1)				
Rifampicin	30 (7.9)				
Clindamycin	269 (71.1)				

**Fig 2:**
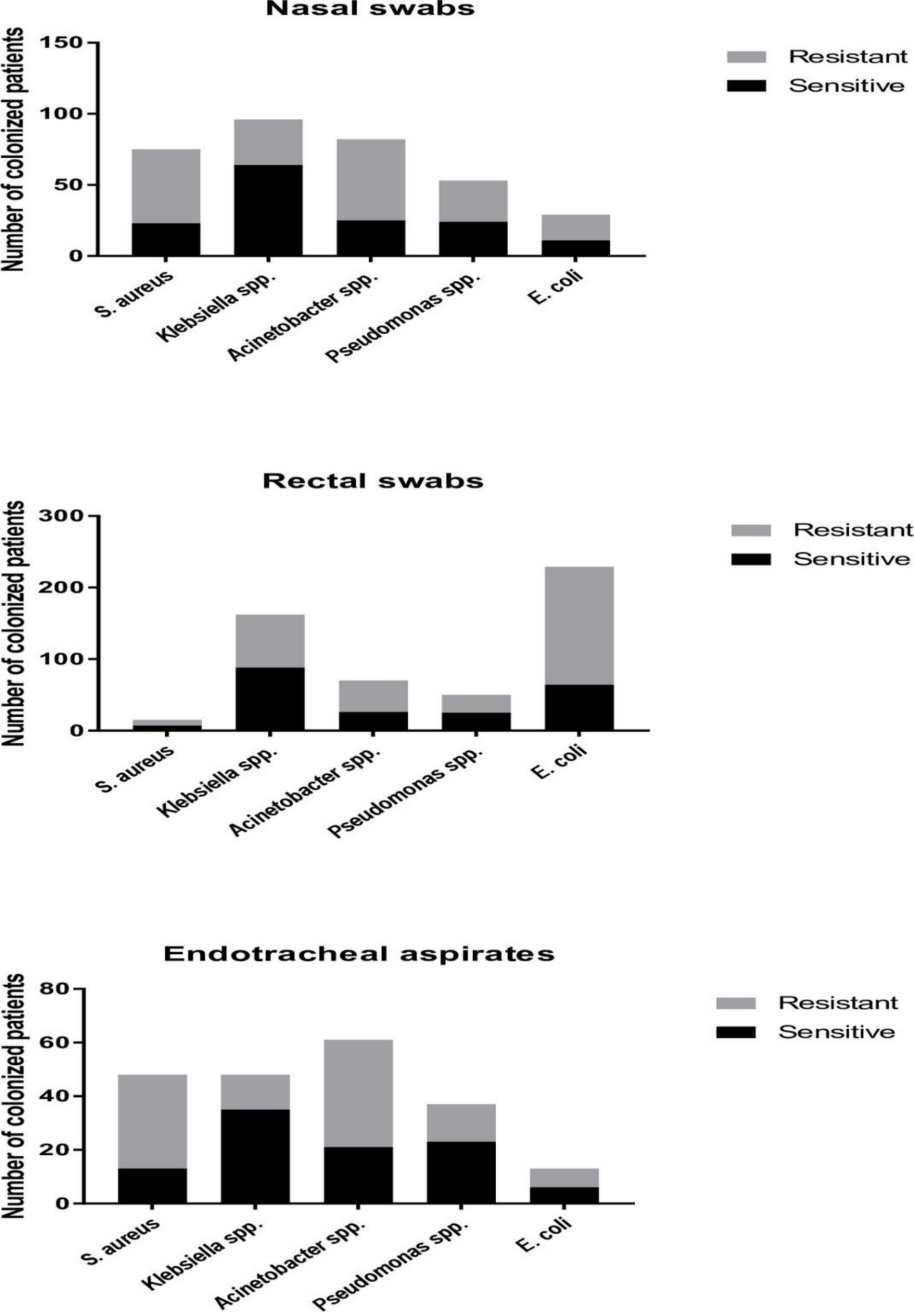
Number of patients became colonized during ICU stay. ICU patients became colonized with sensitive organisms are represented by the black color, and those with AROs are shown by the grey color.

The proportion of patients who acquired either nasal, rectal and/or endotracheal colonization with an ARO was 61.3% (223/364, Fig 2). The 364 patients included in the study represented a total of 2,276 patient days at risk of AROs acquisition in ICU. Therefore, the incidence rate of ICU patients becoming colonized with AROs was 9.8 (223/2,276) per 100 patient days. The acquired AROs colonization rate of the tracheal tract was the highest (61.5%, 80/130), followed by the rectal colonization (54.7%, 199/364) and the nasal colonization (33.8%, 123/364). The overall acquisition risk at any sampling site with an ESBL-producing *E*. *coli* was 39.8% (145/364), carbapenemase-producing *Acinetobacter* spp. 22.0% (80/364), methicillin-resistant *S*. *aureus* 16.2% (59/364), ESBL-producing *Klebsiella* spp. 13.7% (50/364), carbapenemase-producing *Pseudomonas* spp. 11.8% (43/364), carbapenemase-producing *E*. *coli* 3.3% (12/364) and carbapenemase-producing *Klebsiella* spp. 3.3% (12/364). Carbapenem-resistant *Acinetobacter* spp. and methicillin-resistant *S*. *aureus* were the most commonly isolated organisms from nasal swabs, cultured from 15.7% (57/364) and 14.3% (52/364) of patients, respectively. These two organisms were also the most commonly isolated organisms from endotracheal aspirates 30.8% (40/130) and 26.9%, (35/130), respectively. The rate of rectal colonization with 3^rd^-generation cephalosporin-resistant *E*. *coli* among patients was 42.3%, (154/364), followed by 3^rd^-generation cephalosporin-resistant *Klebsiella* spp. (17.3%, 63/364). 124 (34.1%) patients were rectal colonized with ESBL-producing *E*. coli, 51 (14.0%) with ESBL-producing *Klebsiella* spp., and the total acquisition rate of rectal ESBL-producing *Enterobacteriaceae* was 44.2% (161/364).

### Risk factors for acquired colonization during ICU stay

In our univariate analysis (Table 3), we found Charlson Comorbidity Index score as a significant risk factor for acquired colonization with AROs, regardless of anatomical sites (all p-values ≤ 0.01), while receipt of antimicrobial treatment on ICU admission was a significant risk factor for nasal and rectal colonization with AROs (both p-values ≤ 0.001). Admission for tetanus disease reduced the risk of AROs acquisition in nasal and rectal cavity (both p-values ≤ 0.001), and nasogastric tube was associated with reduced risk of rectal AROs colonization (p = 0.01).

**Table 3:** Univariate hazard ratios for risk factors for acquired colonization with AROs, according to Cox regression analysis.

Variables	Nasal colonization (N = 123)		Rectal colonization (N = 199)		Endotracheal colonization (N = 80)	
	HR (95% CI)	p	HR (95% CI)	p	HR (95% CI)	p
Admission for tetanus disease	0.40 (0.27–0.60)	<0.001	0.37 (0.27–0.51)	<0.001	0.63 (0.35–1.13)	0.12
Charlson Comorbidity Index score	1.25 (1.08–1.44)	0.003	1.24 (1.12–1.37)	<0.001	1.45 (1.09–1.93)	0.01
Admission colonization status						
Admission nasal colonization	0.65 (0.37–1.12)	0.12	-	-	-	-
Admission rectal colonization	-	-	1.12 (0.76–1.64)	0.57	-	-
Admission endotracheal colonization	-	-	-	-	0.86 (0.47–1.57)	0.63
Receipt of antimicrobial treatment on admission	1.96 (1.31–2.95)	0.001	1.89 (1.38–2.59)	<0.001	1.44 (0.83–2.51)	0.19
Intensive care procedures on admission						
Nasogastric tube	1.14 (0.79–1.66)	0.48	0.69 (0.51–0.92)	0.01	0.83 (0.52–1.32)	0.42
Respiratory support	1.34 (0.93–1.92)	0.11	-	-	0.91 (0.58–1.43)	0.69

In multivariate analysis (Table 4), hazard ratios did not change much, except for antimicrobial treatment, which was reduced by at least 50% and became non-significant, and Charlson Comorbidity Index score, which was reduced and was no longer a significant risk factor for nasal colonization with AROs. Still, admission for tetanus disease had reduced the risk of nasal and rectal acquisition by AROs, while nasogastric tube reduced the incidence of rectal AROs colonization in ICU.

**Table 4:** Multivariate hazard ratios for risk factors for acquired colonization with AROs, according to Cox regression analysis.

Variables	Nasal colonization (N = 123)		Rectal colonization (N = 199)		Endotracheal colonization (N = 80)	
	HR (95% CI)	p	HR (95% CI)	p	HR (95% CI)	p
Admission for tetanus disease	0.46 (0.29–0.74)	0.001	0.39 (0.27–0.56)	<0.001	0.67 (0.33–1.36)	0.27
Charlson Comorbidity Index score	1.14 (0.97–1.35)	0.12	1.13 (1.01–1.25)	0.04	1.42 (1.03–1.94)	0.03
Admission colonization status						
Admission nasal colonization	0.57 (0.32–1.01)	0.05	-	-	-	-
Admission rectal colonization	-	-	1.19 (0.80–1.76)	0.40	-	-
Admission endotracheal colonization	-	-	-	-	0.76 (0.40–1.45)	0.41
Receipt of antimicrobial treatment on admission	1.28 (0.80–2.04)	0.31	1.19 (0.84–1.71)	0.33	1.20 (0.66–2.18)	0.55
Intensive care procedures on admission						
Nasogastric tube	1.05 (0.68–1.61)	0.83	0.62 (0.46–0.83)	0.002	0.80 (0.48–1.33)	0.40
Respiratory support	1.16 (0.77–1.75)	0.49	-	-	0.82 (0.50–1.35)	0.45

### Characteristics of hospital-acquired infections

**Fig 3:**
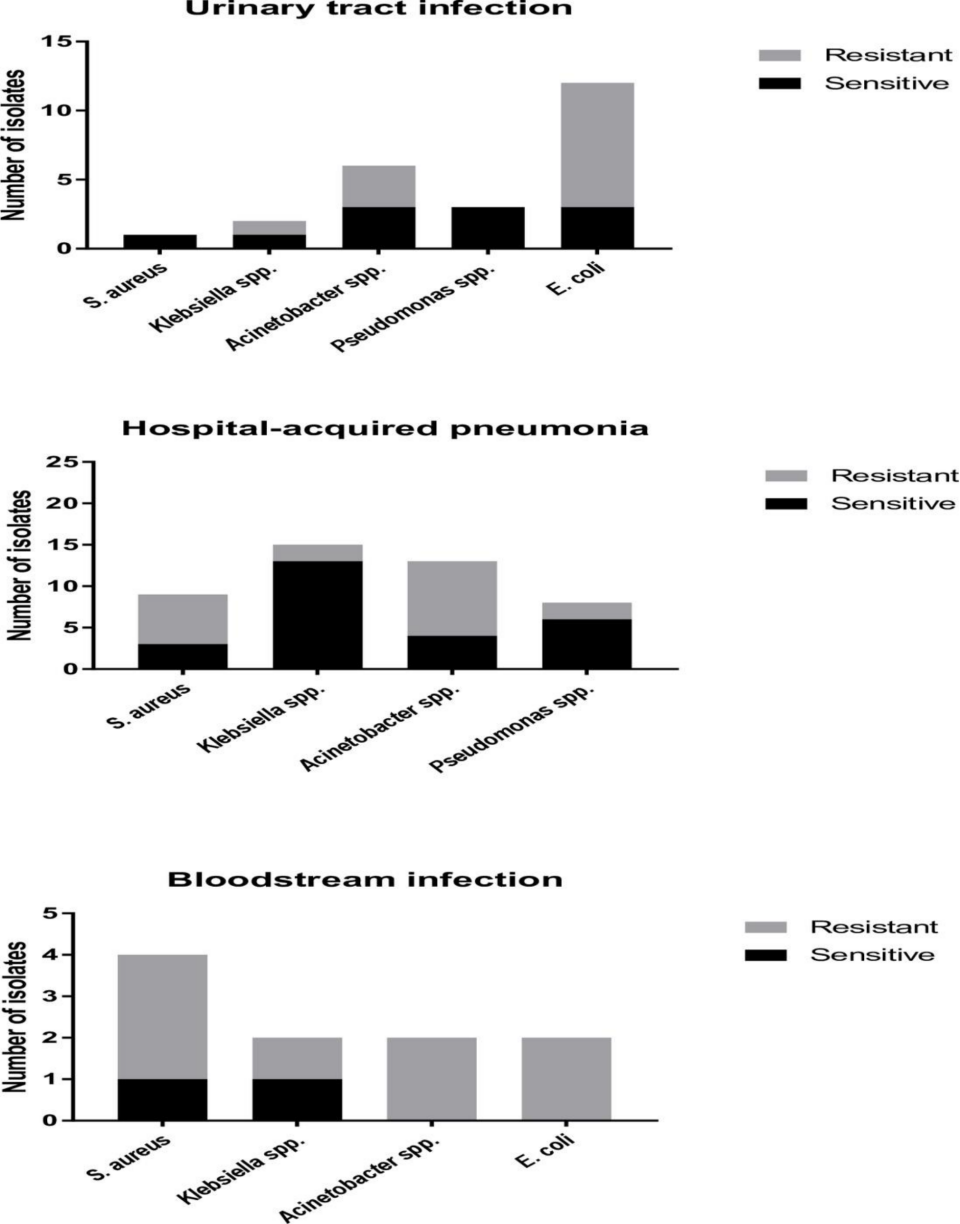
Number of isolates causing hospital-acquired infections. Sensitive pathogens are represented by the black color. AROs are shown by the grey color.

During the study period, 106 episodes of HAIs were observed in 85 patients, of whom 66 patients (77.6%) had a single episode, 18 (21.2%) had two episodes, and 1 (1.2%) had four episodes. The 364 patients included in the study represented a total of 3,701 patient days at risk of contracting HAIs in ICU. Therefore, the proportion of ICU patients with HAIs was 23.4% (85/364), and the incidence rate of ICU patients contracting HAIs was 2.3 (85/3,701) per 100 patients days. The most common type of HAIs were pneumonia (49.1%, 52/106), of which 69.2% (36/52) were ventilator associated infections, followed by urinary tract infections (36.8%, 39/106) and bloodstream infections (14.1%, 15/106). The mean time from admission to the first episode of HAIs was 11.9 ± 6.4 days. Of the 106 episodes of HAIs, 93 (87.7%) were linked to the culture of a single bacterial species and 13 (14.0% of 93) were associated with more than one organism. The distribution of pathogens causing HAIs is shown in Fig 3.

Table 5 shows the relationship between bacterial species of HAIs and prior colonization (either admission colonization or acquired colonization). For example, 100% of the 15 patients with subsequent sensitive *Klebsiella* spp. infections had prior antimicrobial susceptible *Klebsiella* spp. isolated from nasal swabs, rectal swabs and/or endotracheal aspirates. Among the 14 patients who developed carbapenemase-producing *Acinetobacter* spp. infections, 7 (50.0%) had prior colonization with carbapenemase-producing *Acinetobacter* spp.; 6 (66.7%) of the 9 patients with methicillin-resistant *S*. *aureus* infections had prior methicillin-resistant *S*. *aureus* colonization.

**Table 5:** Subsequent hospital-acquired infections and prior colonization (either admission colonization or acquired colonization) with the same organisms among ICU patients.

Bacteria	Hospital-acquired infections (n)	Prior colonization (n, %)	
		Yes	No
**Sensitive *S*. *aureus***	**5**	**2 (40.0%)**	**3 (60.0%)**
**Methicillin-resistant *S*. *aureus***	**9**	**6 (66.7%)**	**3 (33.3%)**
**Sensitive *E*. *coli***	**3**	**3 (100%)**	**0**
**ESBL-producing *E*. *coli***	**8**	**6 (75.0%)**	**2 (25.0%)**
**AmpC-producing *E*. *coli***	**3**	**0**	**3 (100%)**
**Sensitive *Klebsiella* spp.**	**15**	**15 (100%)**	**0**
**ESBL-producing *Klebsiella* spp.**	**1**	**1 (100%)**	**0**
**AmpC-producing *Klebsiella* spp.**	**1**	**1 (100%)**	**0**
**Carbapenemase-producing *Klebsiella* spp.**	**1**	**0**	**1 (100%)**
**Sensitive *Acinetobacter* spp.**	**2**	**1 (50.0%)**	**1 (50.0%)**
**ESBL-producing *Acinetobacter* spp.**	**4**	**1 (25.0%)**	**3 (75.0%)**
**Carbapenemase-producing *Acinetobacter* spp.**	**14**	**7 (50.0%)**	**7 (50.0%)**
**Sensitive *Pseudomonas* spp.**	**9**	**1 (11.1%)**	**8 (88.9%)**
**Carbapenemase-producing *Pseudomonas* spp.**	**2**	**0**	**2 (100%)**
**Total**	**77**	**44 (57.1%)**	**33 (42.9%)**

### Risk factors for hospital-acquired infections

In univariate analysis (Table 6), vascular catheters including central venous, arterial and hemofiltration catheter were found to be a significant risk factor for hospital-acquired bloodstream infection (p = 0.01). Both univariate and multivariate Cox regression analysis (Tables 6 and 7) demonstrated that admission for tetanus disease was a protective factor against the development of hospital-acquired pneumonia in ICU, whereas none of the study factors was significantly associated with HAIs.

**Table 6:** Univariate hazard ratios for risk factors of hospital-acquired infections, according to Cox regression analysis.

Variables	Pneumonia (N = 44)		Urinary tract infection (N = 38)		Bloodstream infection (N = 15)	
	HR (95% CI)	p	HR (95% CI)	p	HR (95% CI)	p
Admission for tetanus disease	0.31 (0.16–0.62)	0.001	1.38 (0.52–5.13)	0.55	0.34 (0.12–1.08)	0.07
Charlson Comorbidity Index score	1.28 (0.92–1.62)	0.13	0.87 (0.37–1.41)	0.66	1.31 (0.86–1.74)	0.17
Prior colonization status						
Prior nasal colonization	0.51 (0.22–1.33)	0.16	-	-	-	-
Prior rectal colonization	-	-	0.49 (0.06–63.59)	0.66	0.52 (0.06–68.40)	0.69
Prior endotracheal colonization	0.77 (0.40–1.55)	0.45	-	-	-	-
Intensive care procedures on admission						
Urinary catheter	-	-	0.83 (0.41–1.64)	0.60	-	-
Respiratory support	1.30 (0.71–2.38)	0.39	-	-	-	-
Vascular catheters	-	-	-	-	5.06 (1.45–15.22)	0.01

**Table 7:** Multivariate hazard ratios for risk factors of hospital-acquired infections, according to Cox regression analysis.

Variables	Pneumonia (N = 44)		Urinary tract infection (N = 38)	
	HR (95% CI)	p	HR (95% CI)	p
Admission for tetanus disease	0.33 (0.16–0.67)	0.002	1.25 (0.41–4.88)	0.72
Charlson Comorbidity Index score	1.04 (0.71–1.53)	0.84	0.93 (0.39–1.47)	0.82
Prior colonization status				
Prior nasal colonization	0.47 (0.16–1.37)	0.17	-	-
Prior rectal colonization	-	-	0.50 (0.06–64.94)	0.67
Prior endotracheal colonization	0.84 (0.37–1.90)	0.69	-	-
Intensive care procedures on admission				
Urinary catheter	-	-	0.85 (0.41–1.68)	0.64
Respiratory support	1.28 (0.65–2.55)	0.48	-	-
Vascular catheters	-	-	-	-

## Discussion

Reports on ICU-acquired bacterial colonization are well-described in high-income settings. To our knowledge, our study is the first prospective longitudinal study to investigate ICU-acquired colonization in Vietnam and one of the few investigating this in a LMIC setting. Our findings show that Vietnam has a high rate of ICU-acquired colonization with AROs (61.3%), especially with methicillin-resistant *S*. *aureus* (16.2%) and ESBL-producing *Enterobacteriaceae* (including 39.8% for *E*. *coli* and 13.7% for *Klebsiella* spp.). The reason behind is multifactorial, but maybe associated with a high proportion of study patients (38.2%) were treated with broad-spectrum antimicrobials within 48 hours of ICU admission which had a negative impact on surveillance culture of all samples. Therefore, more sensitive organisms may not have been detected, resulting in favor of more resistant ones. Moreover, contact isolation or geographic separation of colonized or infected patients with AROs is rarely applied in our unit, so this can contribute to the spread of AROs among ICU patients. Our data can be compared to those from China and France where acquired colonization rates with AROs of 15.2–34.4% have been reported in multicenter studies [26,29].

In terms of HAIs, we found that vascular catheters including central venous, arterial and hemofiltration catheter were the main risk factor for hospital-acquired BSI. This is in accordance with other published data showing vascular catheterization increases the risk of infection because of damaging human natural barriers against infection like skin or mucous membranes. Moreover, contaminated objects or substances may be introduced directly into tissues [30]. Among the 77 patients who developed HAIs with any of the specified bacteria, 44 (57.1%) had prior colonization with the same organism (Table 5), suggesting that prior colonization was an initial stage in the development of HAIs. In our study, we used a combination of bacterial identification on the MALDI-TOF and antimicrobial susceptibility test by disc diffusion method for matching colonized and infected isolates. They are all phenotypic tests, not genotypic methods. Therefore, it is possible that the proportion of matching colonization/infection pairs underestimates the contribution of colonization to infection. In fact, the causal relationship between nasal colonization with *S*. *aureus* and ICU-acquired *S*. *aureus* infections is already well-established [6,7,31,32]. Moreover, the association between gastrointestinal colonization and HAIs caused by *K*. *pneumoniae*, *P*. *aeruginosa* or *A*. *baumannii* is also determined strongly [10,11,33,34]. However, our analyses did not figure out prior colonization was a significant risk factor for HAIs. The explanation may be due to a low number of observed HAIs with specified bacteria in our study. Further understanding of this relationship may be generated through whole-genome sequencing, an approach which would enable a better understanding of the transmission of AROs between patients and over time.

Focusing on early identification of risk factors of acquired AROs colonization may aid in reducing the spread of AROs and subsequent HAIs in ICU settings. Here, Charlson Comorbidity Index score was a significant risk factor for rectal and endotracheal colonization of AROs, and it also seems to increase the risk of nasal colonization with AROs in ICU (HR = 1.14, p = 0.12, Table 4). This is in agreement with some studies conducted in other regions of the world [21,22,24,27,35]. The higher Charlson Comorbidity Index score, the more severe comorbid diseases are existed in human [36]. Indeed, chronic diseases are associated with physical and chemical changes, marked changes occur in the intestinal and respiratory tract which may enhance the risk of bacterial colonization [37,38]. Of note is that admission for tetanus disease reduced significantly the risk of nasal and rectal acquisition by AROs, and probably decreased the risk of endotracheal colonization with AROs (HR = 0.67, Table 4). In our study, all tetanus patients were generally healthy with 83.3% of them having a Charlson Comorbidity Index score of 0. These patients were admitted for primarily control of muscular spasm and therefore may have a lower risk of AROs acquisition than others. This may be a reason why admission for tetanus disease was also a protective factor against the development of hospital-acquired pneumonia in ICU. The other explanation may be related to the tracheostomy procedure being performed in tetanus patients, not intubation technique due to lock-jaw. The tracheostomy tube is placed in the lower airway (below the vocal cords), therefore it is protective against colonization with pathologic organisms from the upper to lower airway [32]. Furthermore, tracheostomy showed significantly less nosocomial pneumonia because of better hygiene and oral care compared to intubation [39]. Additionally, nasogastric tube significantly decreased the risk of acquiring rectal AROs colonization, because all ICU patients with nasogastric tube in our study were fed with enteral nutrition formula produced by the Department of Nutritional of the hospital which can affect the human intestinal tract in ways that might change its hospitality to colonizing or pathogenic bacteria [38]. Remarkably, the association between antimicrobial therapy and the nasal/rectal acquisition of AROs is strong in our study using univariate analysis (Table 3). It is well-known that antimicrobial therapy may promote proliferation of AROs by exerting selective pressure in individual patients, (i.e., inhibition of competing organism but not of resistant organisms) [40]. Once AROs have emerged, antimicrobials may play a crucial role in their subsequent spread from patient to patient [41].

Our study is limited by being conducted in a single tertiary center, which limits generalization of its results to other centers. Many environmental factors, like workload, hand hygiene compliance, room cleaning protocols, and patient-related factors were not evaluated for the risk of acquired colonization and infections. Moreover, we did not investigate the genetic mechanisms of antimicrobial resistance to better understand current resistance and evaluate potential interventions.

## Conclusions

ICU patients are at high risk for acquiring colonization and contracting HAIs, especially with AROs during ICU stay. Although the results show only benefit in a single center, they offer insight for future research which should focus on monitoring colonization, and the development of preventive measures that may halt spread of AROs in ICU settings.

## References

[1] World Health Organization. Report on the Burden of Endemic Health Care-Associated Infection Worldwide. 2011;40.

[2] Ewans TM, Ortiz CR LF. Prevention and control of nosocomial infection in the intensive care unit Intensive Care Med 4th ed Lippincot-Raven, New York 1999;1074–80.

[3] Elston J, Hinitt I, Batson S, Noakes C, Wright J, Walley J, et al Infection control in a developing world. 2013;67(10):45–50. 24397225

[4] Phu VD, Wertheim HFL, Larsson M, Nadjm B, Dinh QD, Nilsson LE, et al Burden of hospital acquired infections and antimicrobial use in Vietnamese adult intensive care units. PLoS One. 2016;11(1):1–15. 10.1371/journal.pone.0147544 PMC4732823 26824228

[5] Phu VD, Nadjm B, Hoang N, Duy A, Co DX, Thi N, et al Ventilator-associated respiratory infection in a resource-restricted setting: impact and etiology. J Intensive Care. 2017;5:1–9. 10.1186/s40560-016-0195-7 29276607 PMC5738227

[6] Niven DJ, Laupland KB, Gregson DB, Church DL. Epidemiology of Staphylococcus aureus nasal colonization and influence on outcome in the critically ill. J Crit Care. 2009;24(4):583–9. 10.1016/j.jcrc.2008.10.004 19327313

[7] Ajao AO, Harris AD, Johnson JK, Roghmann M, Perencevich EN, Schweizer ML, et al Association between Methicillin-Resistant Staphylococcus aureus Colonization and Infection May Not Differ by Age Group. Infect Control Hosp Epidemiol. 2009;3:9–11. 10.1086/668773 PMC3677581 23221199

[8] Galoisy-Guibal L, Soubirou JL, Desjeux G, Dusseau JY, Eve O, Escarment J, et al Screening for multidrug-resistant bacteria as a predictive test for subsequent onset of nosocomial infection. Infect Control Hosp Epidemiol. 2006;27(11):1233–41. 10.1086/507277 17080382

[9] Dickstein Y, Edelman R, Dror T, Hussein K, Bar-Lavie Y, Paul M. Carbapenem-resistant Enterobacteriaceae colonization and infection in critically ill patients: a retrospective matched cohort comparison with non-carriers. J Hosp Infect. 2016;94(1):54–9. 10.1016/j.jhin.2016.05.018 27392978

[10] Gómez-Zorrilla S, Camoez M, Tubau F, Cañizares R, Periche E, Dominguez MA, et al Prospective Observational Study of Prior Rectal Colonization Status as a Predictor for Subsequent Development of Pseudomonas aeruginosa Clinical Infections. Antimicrob Agents Chemother. 2015;59(9):5213–9. 10.1128/AAC.04636-14 26077248 PMC4538513

[11] Corbella X, Pujol M, Ayats J, Sendra M, Ardanuy C, Domínguez M a, et al Relevance of digestive tract colonization in the epidemiology of nosocomial infections due to multiresistant Acinetobacter baumannii. Clin Infect Dis. 1996;23(2):329–34. 8842272 10.1093/clinids/23.2.329

[12] Nguyen K Van Thi Do NT, Chandna A Nguyen TV, Pham C Van Doan PM, et al Antibiotic use and resistance in emerging economies: a situation analysis for Viet Nam. BMC Public Health. 2013;13(1):1158. 24325208 10.1186/1471-2458-13-1158 PMC4116647

[13] Nga DTT, Chuc NTK, Hoa NP, Hoa NQ, Nguyen NTT, Loan HT, et al Antibiotic sales in rural and urban pharmacies in northern Vietnam: an observational study. BMC Pharmacol Toxicol. 15(1):6. 24555709 10.1186/2050-6511-15-6 PMC3946644

[14] Thuy DB, Campbell J, Hoang NVM, Thi T, Trinh T, Thi H, et al A one-year prospective study of colonization with antimicrobial-resistant organisms on admission to a Vietnamese intensive care unit. PLoS One. 2017;1–8. 10.1371/journal.pone.0184847 PMC5599024 28910379

[15] Clinical and Laboratory Standards Institute. M100-S25 Performance Standards for Antimicrobial Susceptibility Testing; Twenty-Fifth Informational Supplement. 2015.

[16] Centers for Disease Control and Prevention. CDC / NHSN Surveillance Definitions for Specific Types of Infections. 2014;

[17] Farrar JJ, Yen LM, Cook T, Fairweather N, Binh N, Parry J, et al Tetanus. EMBO J. 2000;292–301. 10.1136/jnnp.69.3.292 PMC1737078 10945801

[18] Balk RA. Systemic inflammatory response syndrome (SIRS). Where did it come from and is it still relevant today? Virulence. 2014;5(1):20–6. 10.4161/viru.27135 24280933 PMC3916374

[19] Singer M, Deutschman CS, Seymour CW, Shankar-Hari M, Annane D, Bauer M, et al The Third International Consensus Definitions for Sepsis and Septic Shock (Sepsis-3). JAMA. 2016;315(8):801–10. 10.1001/jama.2016.0287 26903338 PMC4968574

[20] Yee F, Singh N, Gayowski T, Wagener MM, Marino IR. Staphylococcus aureus Nasal Colonization in Patients with Cirrhosis: Prospective Assessment of Association with Infection. Infect Control Hosp Epidemiol. 1998;19(5):328–32. 9613693 10.1086/647823

[21] Safdar N, Maki DG. The Commonality of Risk Factors for Nosocomial Colonization and Infection with Antimicrobial-Resistant Staphylococcus aureus, Enterococcus, Gram-Negative Bacilli, Clostridium difficile, and Candida. Am Soc Intern Med. 2002;136:834–44. 10.7326/0003-4819-136-11-200206040-00013 12044132

[22] Corea E, Silva T De, Perera J. Methicillin-resistant Staphylococcus aureus: prevalence, incidence and risk factors associated with colonization in Sri Lanka. J Hosp Infect. 2003;6701:145–8. 10.1016/s0195-6701(03)00256-1 14529641

[23] Nseir S, Blazejewski C, Lubret R, Wallet F, Courcol R, Durocher a. Risk of acquiring multidrug-resistant Gram-negative bacilli from prior room occupants in the intensive care unit. Clin Microbiol Infect. 2011;17(8):1201–8. 10.1111/j.1469-0691.2010.03420.x 21054665

[24] Mittal G, Gaind R, Kumar D, Kaushik G, Gupta KB, Verma PK, et al Risk factors for fecal carriage of carbapenemase producing Enterobacteriaceae among intensive care unit patients from a tertiary care center in India. BMC Microbiol. 2016;16(138):1–10. 27392139 10.1186/s12866-016-0763-y PMC4938945

[25] Nakai H, Hagihara M, Kato H, Hirai J, Nishiyama N. Prevalence and risk factors of infections caused by extended-spectrum b-lactamase (ESBL)-producing Enterobacteriaceae. J Infect Chemother. 2016;22(5):319–26. 10.1016/j.jiac.2016.02.004 26968486

[26] Masse J, Elkalioubie A, Blazejewski C, Ledoux G, Wallet F, Poissy J. Colonization pressure as a risk factor of ICU-acquired multidrug resistant bacteria: a prospective observational study. Eur J Clin Microbiol Infect Dis. 2017;36:797–805. 10.1007/s10096-016-2863-x 28000030

[27] Detsis M, Karanika S, Mylonakis E. ICU Acquisition Rate, Risk Factors, and Clinical Significance of Digestive Tract Colonization With Extended-Spectrum Beta-Lactamase–Producing Enterobacteriaceae: A Systematic Review and Meta-Analysis*. Crit Care Med. 2017;45(4):705–14. 10.1097/CCM.0000000000002253 28157141

[28] Cronin KM, Lorenzo YSP, Olenski ME, Bloch AE, Visvanathan K, Waters MJ, et al Risk factors for KPC-producing Enterobacteriaceae acquisition and infection in a healthcare setting with possible local transmission: a case control study. J Hosp Infect. 2017;96(2):111–5. 10.1016/j.jhin.2017.02.010 28389093

[29] Ma X, Wu Y, Li L, Xu Q, Hu B, Ni Y, et al First multicenter study on multidrug resistant bacteria carriage in Chinese ICUs. BMC Infect Dis. 2015;15(1):358. 26290050 10.1186/s12879-015-1105-7 PMC4545921

[30] Ducel G., Fabry LN J. Prevention of hospital-acquired infections. World Heal Organ. 2002;1–64.

[31] Honda H, Krauss MJ, Coopersmith CM, Kollef MH, Richmond AM, Fraser VJ, et al Staphylococcus aureus Nasal Colonization and Subsequent Infection in Intensive Care Unit Patients: Does Methicillin Resistance Matter? Infect Control Hosp Epidemiol. 2010;31(6):584–91. 10.1086/652530 20426656 PMC4154586

[32] Kao Kuo-Chin, Chen Chun-Bing, Hu Han-Chung, Chang Hui-Ching, Huang Chung-Chi and Y-CH. Risk Factors of Methicillin-Resistant Staphylococcus aureus Infection and Correlation With Nasal Colonization Based on Molecular Genotyping in Medical Intensive Care Units. Medicine (Baltimore). 2015;94(28):1–7. 10.1097/MD.0000000000001100 PMC4617090 26181545

[33] Gorrie CL, Mirceta M, Wick RR, Edwards DJ, Thomson NR, Strugnell RA, et al Gastrointestinal carriage is a major reservoir of K. pneumoniae infection in intensive care patients. Clin Infect Dis. 2017;00(00):1–8. 10.1093/cid/cix270 PMC5850561 28369261

[34] Martin RM, Bachman MA. Colonization, Infection, and the Accessory Genome of Klebsiella pneumoniae. Front Cell Infect Microbiol. 2018;8(4):1–15. 29404282 10.3389/fcimb.2018.00004 PMC5786545

[35] Selden R, Lee S, Lan WEN, Wang LOU, Ph D. Nosocomial Klebsiella Infections: Intestinal Colonization as a Reservoir. Ann Intern Med. 1971;74(5):657–64. 5559431 10.7326/0003-4819-74-5-657

[36] Charlson ME, Pompei P, Ales KL, MacKenzie R. A new method of classifying prognostic in longitudinal studies: development and validation. Vol. 40, Journal of Chronic Diseases. 1987 p. 373–83. 3558716 10.1016/0021-9681(87)90171-8

[37] Siegel SJ, Weiser JN. Mechanisms of Bacterial Colonization of the Respiratory Tract. Annu Rev Microbiol. 2015;425–44. 10.1146/annurev-micro-091014-104209 26488280 PMC4760621

[38] Kirkland KB. Bacterial Colonization: Can We Live With It? Clin Infect Dis. 2009;48(10):1382–4. 10.1086/598195 19348595

[39] Rumbak MJ, Newton M, Truncale T, Schwartz SW, Adams JW, Hazard PB. A prospective, randomized, study comparing early percutaneous dilational tracheotomy to prolonged translaryngeal intubation (delayed tracheotomy) in critically ill medical patients. Crit Care Med. 2004;32(8):1689–94. 15286545 10.1097/01.ccm.0000134835.05161.b6

[40] Donskey CJ. Antibiotic Regimens and Intestinal Colonization with Antibiotic-Resistant Gram-Negative Bacilli. Clin Infect Dis. 2006;43:S62–9. 10.1086/504481 16894517

[41] Donskey CJ. The Role of the Intestinal Tract as a Reservoir and Source for Transmission of Nosocomial Pathogens. 2004;39:219–26. 10.1086/422002 15307031

